# Challenges and Improvements of Novel Therapies for Ischemic Stroke

**DOI:** 10.3389/fphar.2021.721156

**Published:** 2021-09-30

**Authors:** Lijun Yang, Jing Qian, Bo Yang, Qiaojun He, Jiajia Wang, Qinjie Weng

**Affiliations:** ^1^ Center for Drug Safety Evaluation and Research, Zhejiang Province Key Laboratory of Anti-Cancer Drug Research, College of Pharmaceutical Sciences, Zhejiang University, Hangzhou, China; ^2^ Zhejiang Center for Drug and Cosmetic Evaluation, Hangzhou, China; ^3^ Department of Cardiology, The Second Affiliated Hospital, School of Medicine, Zhejiang University, Hangzhou, China

**Keywords:** ischemic stroke, neuroprotective agent, advances and limitations, structure transformation, combination therapy

## Abstract

Stroke is the third most common disease all over the world, which is regarded as a hotspot in medical research because of its high mortality and morbidity. Stroke, especially ischemic stroke, causes severe neural cell death, and no effective therapy is currently available for neuroregeneration after stroke. Although many therapies have been shown to be effective in preclinical studies of ischemic stroke, almost none of them passed clinical trials, and the reasons for most failures have not been well identified. In this review, we focus on several novel methods, such as traditional Chinese medicine, stem cell therapy, and exosomes that have not been used for ischemic stroke till recent decades. We summarize the proposed basic mechanisms underlying these therapies and related clinical results, discussing advantages and current limitations for each therapy emphatically. Based on the limitations such as side effects, narrow therapeutic window, and less accumulation at the injury region, structure transformation and drug combination are subsequently applied, providing a deep understanding to develop effective treatment strategies for ischemic stroke in the near future.

## Introduction

Stroke, a kind of cerebrovascular accident, is a public-concerning health problem all over the world, ranking only next to cardiovascular diseases and cancer (Ouk et al., 2019). Stroke is the second leading cause of death with mortality around 25% and the third cause of disability with a morbidity rate around 5% (Schnabel et al., 2019; Campbell and Khatri, 2020). Ischemic stroke caused by vascular obstruction accounts for 80% of all strokes (Donnan et al., 2008). Although many factors, including overweight, binge drinking, high blood pressure, smoking, and COVID-19 can raise the risk for stroke, the risk factor for about 30% of patients with ischemic stroke is still unknown (Boehme et al., 2017). Moreover, most patients can recover from ischemic stroke due to effective treatments; however, the majority of them have sequelae such as cognitive impairment, hemiplegia, depression, swallowing disorder, and language barrier (Shi et al., 2015). Besides, the recurrence risk of stroke is quite high, and the recurrence reaches 30% at 5 years (Schnabel et al., 2019).

**FIGURE 1:**
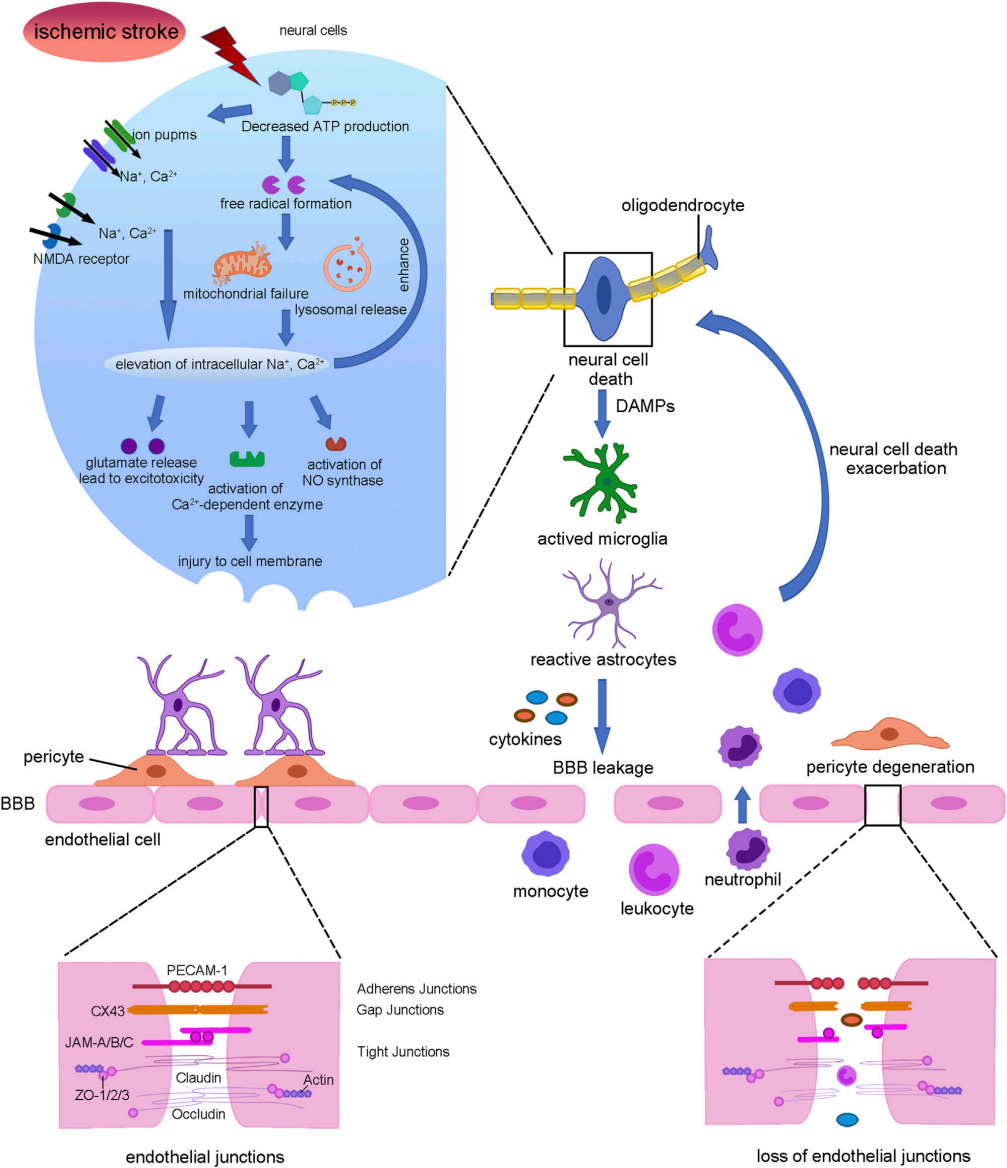
Summary of stroke pathophysiology. Interruption of blood flow leads to neural cell death which release DAMPs to activate microglia and astrocytes which secreting cytokines. Then pericyte was degenerated and endothelial tight junctions were lost, leading to BBB leakage. Further, monocyte, leukocyte, and neutrophil infiltration from blood exacerbated neural cell death and demyelination.

Ischemic stroke is thought to be caused by impaired blood flow, which brings out an ischemic cascade and then leads to intensive neuronal injury (Koh and Park, 2017) (Figure 1). At the initial acute stage, the reduced blood flow leads to the failure of high energy metabolism in neural cells, blocking the ATP synthesis. As a result, the activity of ion pumps and NMDA receptors is inhibited, causing intracellular accumulation of Ca^2+^, Na^+^, and Cl^−^ (Volz et al., 2020). In addition, ATP reduction also causes the lysis of mitochondrion and lysosome, which hydrolyzes intracellular materials and further promotes calcium influx (Pradeep et al., 2012). Then increased calcium concentrations result in the releasing of the excitatory amino acid, glutamate, which eventually becomes excitotoxicity (Ge et al., 2020). Apart from that, calcium influx activates nitric oxide synthase and calcium-dependent enzymes such as protein kinase C and phospholipase A2, aggravating cell death (Jiang et al., 2018). In addition, the injured neural cells produce and release free radicals, reactive oxygen species (ROS), and danger-associated molecular patterns (DAMPs). Then DAMPs activate microglia and astrocytes to enhance the release of inflammatory factors, thus causing leukocytes and white blood cells recruitment and adherence to endothelial cells. As a consequence, the endothelial cytoskeleton alters and pericytes degenerate, accompanied by enzymatic cleavage of endothelial junction proteins including adherens junctions, gap junctions, and tight junctions, resulting in a disrupted blood–brain barrier (BBB) subsequently. In turn, the leak of the BBB leads to pro-inflammation cytokines accumulation at the injured site, exacerbating neural cell and oligodendrocytes death (Rodgers et al., 2015).

Currently, tissue plasminogen activator (tPA), a thrombolytic agent to restore blood flow, is the only drug used in the clinical treatment of stroke approved by FDA, but only 10% of patients meet the standard for using tPA as a therapeutic drug for stroke (Fugate and Rabinstein, 2014). Another way to treat ischemic stroke is surgery; however, not all patients can take the surgery if their carotid arteries are mostly blocked, and surgery may increase the risk of disability (Yang et al., 2020). Hence, the rapid neural cell death, the limited spontaneous functional recovery, and the lack of therapeutic methods make it quite difficult to protect from ischemic stroke. Nowadays, a rapidly developing field of stroke recovery studies indicates that traditional Chinese medicine (TCM), stem cells, and exosomes regulate the expression of various proteins and factors after stroke to reduce infract volume (Larpthaveesarp et al., 2021). Meanwhile, stem cells could differentiate into glial cells to replace the necrotic cells and the exosomes contains various cargos, both of which have effects on ameliorating the motor functional recovery after stroke (Han et al., 2021). TCM has been used in Eastern Asia for ischemic stroke treatment because of its function in preventing brain edema, neuronal apoptosis, and improving microenvironment. Thus, these promising therapies may have significant impacts on stroke treatment.

Usage of tPA in stroke treatment is worldwide; however, the therapeutic window is quite narrow, can only be applied within 4.5 h after stroke, and might come along with severe side effects such as hemorrhage (Wardlaw et al., 2014). Other drugs such as aspirin, alteplase, rosuvastatin, and warfarin are used in stroke therapy, but all of them are etiological treatment. However, some factors affect these therapie’s application, such as immune transplant rejection, side effect, unknown mechanism, and the risk of tumor formation or the promising sources, so many approaches have been developed to solve these problems (Donders et al., 2015). Structure transformation is one of the important ways, and labeling cells with iron chains makes it trackable and enhances the homing to the infraction area (Zhang et al., 2019). Moreover, drug combination also helps to extend the therapeutic window, enhance the neuroprotective effect, and reduce the side effects of some drugs although these novel therapies are still under clinical trials, indicating that there are many ways to improve the limits of novel therapies.

This review briefly discussed stroke and the ischemic cascades which caused cell death in stroke. We mainly focused on the advancements and current limitations of new therapeutic methods including TCM and stem cells as well as exosomes; we also discussed their potential mechanisms. Based on the limitations, we discussed the renovation of the structure of TCM, cells, and exosomes, providing ideas for how to improve the effectiveness and safety of these agents. Also, we talked about their known preclinical and clinical curative effects as well as drug combination therapy, providing new insights in stroke therapy.

## Traditional Chinese Medicine Therapy

TCMs have been developed for thousand years and almost all of them are based on the Compendium of Materia Medica and Huangdi Neijing. Nowadays, many countries use TCM as a therapeutic method in fever, dementia, hepatitis, insomnia, myocardial infarction, and other diseases. TCM is made up of plant or animal elements; sometimes human and mineral production can also be used as a TCM. Further, several TCM compounds have been approved by the National Medical Products Administration (NMPA) for the treatment of stroke. In this section, we will focus on the therapeutic effect of TCM, the active components, their potential mechanism, and their limitations and improvements.

### Molecular Mechanisms of Traditional Chinese Medicine Therapy

Drugs such as Shuxuening injection, Ginaton, and extract of *Ginkgo biloba* leave’s tablets, whose main active ingredients are ginkgolides and bilobalide, have shown neuroprotective effect in ischemic stroke. Ginkgolides-injected mice showed less infarct area and mortality, decreased motor disorders frequency, and mortality. On the one hand, ginkgolides treatment reduced the BBB permeability, with increased Zonula occludens-1 (ZO-1) and less brain edema (Wang et al., 2014). On the other hand, ginkgolides also have neuron protection effect, with less neuron apoptosis indicated by decreased cleaved caspase-3, attenuated shrinkage, and atrophy of the neurons (Ma et al., 2012). Bedsides, the level of ROS was diminished after ginkgolides administration, accompanied by decreased malondialdehyde (MDA), nitric oxide (NO), and nitric oxide synthase (NOS) and increased superoxide dismutase (SOD) (Cui et al., 2020). Inflammation is crucial for stroke recovery as it accelerates stroke damage, and ginkgolides have effect on inhibition of inflammation. It has been reported that injecting ginkgolides after MCAO could suppress the expression of tumor necrosis factor α (TNF-α) and interleukin 1β (IL-1β) while promoting the expression of IL-10 and switch microglia into anti-inflammatory M2 phenotype (Cui et al., 2020). In addition, through activation of Janus Kinase/signal transducer and the activator of transcription 3 (JAK/STAT3) pathway, ginkgolides upregulate HIF-1α and vascular endothelial growth factor (VEGF) which improved angiogenesis (Chen et al., 2018a). At the beginning of stroke, hypoxia induces anaerobic respiration, thus enhancing glutamate, glycine, and aspartic acid and finally leading to excitotoxicity; however, pre-administrating mice with ginkgolides meliorated this progress (Yang et al., 2011).

Another effect agent used in TCM for stroke is Salvianolate (Sal), which is frequently applied in NMPA-approved drugs such as *Salviae miltiorrhizae* and Ligustrazine Hydrochloride Injection, Danhong Injection, Fufang Danshen Injection, and Salvianolate injection. Treatment of Sal ameliorated cognitive deficits, the stroke area, motor impairments, and memory deficits (Luan et al., 2020). Sal could prevent cells from ROS by increasing the expression of nuclear factor e2-related factor 2 (Nrf2) and Heme oxygenase-1 (HO-1) *in vivo* and *in vitro* H_2_O_2_-treated neuron cells combined with Sal had better cell survival (Zhang et al., 2012). Sal remarkably reduced the permeability of BBB since the Evans blue extravasation was decreased and the tight junction proteins ZO-1, occludin, and claudin-5 were increased; *in vitro* studies showed that treating endothelial cells with Sal ameliorated the reduced level of Transepithelial electrical resistance (TEER) which was induced by oxygen-glucose deprivation (OGD), indicating the integrity of the BBB (Liu et al., 2021a; Yuan et al., 2021). Then, mitochondria damage was also attenuated by Sal through activating the PI3K/AKT pathway *in vivo*; transmission electron microscopy showed that the number of swollen and destroyed mitochondria was decreased after Sal treatment, and Rhodamine 123 staining showed more matrix metallopeptidase (MMP) positive cells (Hou et al., 2016). Additionally, Sal also plays a role in promoting angiogenesis in the stroke model. There were more microvessels in the infarct area and BrdU staining showed more formation of vessels, meanwhile the proliferation, recruitment, and coverage of pericytes around vessels were increased (Lyu et al., 2017). Sal injection promoted the level of Golgi matrix protein GM130 and maintained the structure of Golgi as a result the mortality of neural cells was reduced in the stroke model (Wang et al., 2013). Apart from these, Sal could rescue stroke *via* regulating glutamate metabolism, neurotrophic factors, and growth factors (Chien et al., 2016).

The other frequently used TCMs in stroke are Xue-Sai-Tong, Xue Shuan tong, Sanqi Tongshu capsule, and San Qi Zong Dai Pian and their main sharing ingredient is *Panax notoginseng* Saponins (PNS). Compared with the control group, PNS reduced the infarction area and increased Nissl bodies markedly; besides, the TXA_2_ (platelet activator)/PGI_2_ (platelet inhibitor) ratio, the indictor for vascular tension, and gastrointestinal bleeding were reduced. Meanwhile, PNS led to fewer calcium ions influx and more cAMP by binding to Glycoprotein 1b platelet subunit alpha (GP1BA), which prevented the formation and exacerbation of platelet aggregation (Xu et al., 2021). In addition, PNS had effects on neuron protection through inhibiting apoptosis and neural plasticity–related protein and promoting cell viability, NSCs proliferation and differentiation, and neurotrophic factors secretion (Wu et al., 2020). For one thing, treatment of PNS could downregulate caspase 1/3/8 as well as TUNEL-positive cells, and *in vitro* study also showed improved cell viability and morphology with increased EGFR/PI3K/AKT expression in SH-SY5Y cells (Li et al., 2009). For another, neuron cell was rescued by PNS *via* decreasing the level of neural plasticity-associated proteins such as Nogo-A, NgR, and neurotrophic factor Rho–associated protein kinase 2 (ROCK2) (Shi et al., 2016). Apart from that, the percentage of NSCs and astrocytes were upregulated and the death of astrocytes was diminished after PNS treating. Additionally, the anti-inflammation effects of PNS helped mice recover from ischemic stroke by reducing TNF-α and IL-6 through activating NF-κB and inhibiting miR155 (Meng et al., 2019). In addition, PNS acted on death-associated protein kinase 1 (DAPK1) to inhibit Ser-1303 phosphorylation in an Anti-N-methyl-d-aspartate receptor (NMDAR), blocking the overactivation of NMDAR against excitotoxicity induced by stroke (Zhang et al., 2020). At the same time, PNS also played an important role in regulating ROS, mitochondria stress, and cerebrovascular reperfusion in rescuing the motor function, the infarct area, and functional recovery in stroke.

Apart from these active components mentioned, there are still many active ingredients that are effective in the treatment of stroke in preclinical studies and are under clinical studies (Supplementary Table S1). For example, breviscapine could alleviate cognitive impairments and neuro defects caused by stroke through its anti-inflammatory and antioxidant, promoting autophagy (Li et al., 2020). Timosaponin B and scutellarein had neuroprotective effects against stroke by their antithrombotic, antiplatelet, and anticoagulation effects (Dong et al., 2019). Shikonin ameliorated BBB permeability, attenuated inflammation, and ROS, leading to experimental stroke protection (Wang et al., 2010).

### Current Limitations for Traditional Chinese Medicine Therapy

The most important weakness of TCM is that the specific mechanism is unsure, as its basic concept is called Qi which is thought surges through the whole body. Other professional terms used in TCM such as yin-yang, wuxing, xue, zang-fu, Jing Luo are invisible and cannot be fully understood by most people, and in TCM, it is recognized that a disease is caused by the imbalance between yin and yang. Different TCMs were used to restore the balance through specific treatment to people in different stage (Xiao and Luo, 2018). What is more, nowadays more people choose to use TCM as a therapeutic way leading to a dramatic increase in demand, so the quality may not reach the standard. Except for some non-harmful materials in TCM, the environmental pollution, the use of pesticides, fertilizers, and the supplement of additives, caused the contamination of TCM. Use of fake medicines instead of TCM makes matter worse, leading to some TCM recalled by FDA since 2004 (Tang et al., 2018).

In addition, TCM administration may cause adverse events. 29 clinical studies reported side effects for salvianolate in stroke treatment, including dizziness and hemorrhage of digestive tract or skin as well as mucosa, liver, or kidney injury (Xin et al., 2020). *Periploca forrestii* Schltr treatment led to 22 patients with drug-induced liver injury (Feng et al., 2016). At the same time, nephrotoxicity such as acute kidney toxicity, nephrolithiasis, urothelial cancer, and rhabdomyolysis was detected after TCM treatment (Yang et al., 2018). In recent days, liver and kidney injury have become the most common adverse events because TCM was metabolized through them but the mechanism is unknown.

Additionally, some herbs have inner toxicity such as cardiologic and neurologic toxicity which may harm patients. *Aconitum carmichaelii* was used for anti-inflammation; however, a hundred cases of aconitine alkaloids toxicity had been reported, most of which had cardiac toxicity that may cause by ion channel disorders and mitochondrial-mediated cell apoptosis (Zong et al., 2019). Besides, neurologic toxicity like seizures and paresthesia would be caused by aconitine alkaloids treatment. High dose or long-term administration PNS would lead to drop in heart rate, left ventricular systolic pressure, and arterial pressure reduction, and in severe cases, it could even lead to bradycardia, atrioventricular block, and even death. Realgar contained arsenic trioxide, which had been identified as a cytoplasmic poison in current studies, and when more than a safety dose was used for neuronal therapy, brain swelling, cerebral hemorrhage, hypoxia, or other neurological toxicity occur.

### Improvements for Traditional Chinese Medicine Therapy

It is still hard to distinguish whether the TCM are better than their active ingredients or not in stroke treatment. On the one hand, Huatuo Zaizao Wan and Zhong Feng Hui Chun Pian, the formulation of TCM is acquired from Compendium of Materia Medica which shows obvious ameliorated stroke symptoms upon the TCM treatment, but the active ingredients have not been figured out yet (Duan et al., 2017). Apart from the active ingredients, there are several pharmaceutical excipients that contribute to promote absorption of drugs, increase the transmission of active ingredients to the disease region, and reduce active ingredients degradation. Thus, TCM may have better therapeutic effects than their active ingredients. As shown by Xiao et al., both Shuxuening injection and its active ingredient ginkgolides reduced the cerebral infarct size, edema, and a neurological deficit score in the MCAO mice model. However, mice treated with Shuxuening injection recovered better than mice treated with ginkgolides (Xiao et al., 2019). On the other hand, the extracted active ingredients have definite pharmacological effects as well as a higher purity than the original TCM. Purity is the key factor to its efficacy and the quality of the extracted active ingredients is easy to control, which helps to reduce the drug toxicity and improve therapeutic effects. For example, the recovery of cognitive and motor functions in patients having Sal was 1.23-fold higher than those taking Danshen injection (Zhang et al., 2018). Similarly, PNS increased the proliferation, invasion, and tube formation of endothelial cells *in vitro* and blood vessel formation in zebrafish, showing a better effect than *Panax notoginseng* injection (Hong et al., 2009; Zhang et al., 2018).

In order to make sure the quality of a drug and reduce its inner toxicity, people analyze the structure of the active ingredient and then modify and synthesize it artificially. Dl-3-n-Butylphthalide (NBP) is a synthetic chiral drug and its prototype compound is isolated from *Apium graveolens* seeds. Till 1995, NBP was found to be useful for ischemic stroke and later experiments showed that both injecting NBP 3 h before or 2 h after surgery had improvement on infarction volume and motor functional recovery, and clinical studies showed that patients using NBP alone had better neurological function than control groups; however, there was no significant difference for the efficacy between NBP and tPA, so NBP had been used for stroke treatment in China (Xu et al., 2019). Anti-inflammation was firstly found in the NBP-treated stroke model; NBP enhanced the proliferation of microglia and promoted the microglia polarization into M2 phenotype, an anti-inflammation phenotype, by activating the calcium/calmodulin-dependent protein kinase β (CaMKKβ)-AMPK pathway or PPARγ nuclear translocation; NBP also decreased the infiltration of endothelial cells by downregulating intercellular adhesion molecule 1 (ICAM-1) and protease-activated receptor-1 (PAR-1) (Zeng et al., 2020; Liu et al., 2021b). In addition, NBP played a role in promoting neuroplasticity, neurite outgrowth, and inhibiting neuron apoptosis, and the NgR, Mogo-A, and caspase 3 were decreased and growth factors such as BDNF, NGF, and neurite branches were increased (Zhang et al., 2021).

tPA is the only FDA approved drug for stroke treatment, but the narrow time window and the risk for hemorrhage limited its clinical use; however, a TCM compound called T541, which consist of Astragalus saponins, salvianolic acids, and PNS, injected together with tPA could attenuate hemorrhage and angioedema caused by tPA, mainly because that T541 could increase BBB integrity and upregulate energy metabolism, thus increasing the fibrinolytic ability and extended the time window for tPA (Chen et al., 2018b). Except tPA, cells could interact with TCM to protect and recover ischemic stroke better. Some studies showed that injecting tetramethylpyrazine (TMP) 2 h post MCAO followed by MSCs intravenous 20 h later would promote the migration of MSCs to the infarction area, and even pretreated MSCs with TMP for 24 h before injection still increased the homing to the infarction area, leading to neurogenesis and angiogenesis improvement through upregulating CXCR4 and SDF-1 (Li et al., 2019). Besides, Zhao et al. showed that TCM such as PNS, Astragalus, and Ren Shen significantly augmented the differentiation of MSCs into neuron-like cells as well as led MSCs from peripheral to the stroke region, helping to remove blood stasis and stimulate neogenesis (Zhao et al., 2012). Meanwhile, MSCs combined with NBP and sodium ferulate helped to increase the secretion of trophic factors such as VEGF and BDNF by astrocytes also enhanced the vessel tube formation by increasing AKT/mTOR (Zhang et al., 2017a).

## Stem Cell Therapy

MSCs and NSCs are stem cells that maintain self-renewal and multipotent ability, and they can differentiate into variety of cell types, such as osteoblasts, adipocytes, and oligodendrocytes as well as neuron-like cells (Beattie and Hippenmeyer, 2017). For their high multilineage differentiation, easily isolated and grafted, raising no ethical concern, and minimal immunoreactivity, stem cells transplantation has become a hotspot in stroke therapy. Compared with the control group, increased proliferation of neurons and microglia is observed when we treated stroke rats with NSCs 1 week or even 4 weeks after the surgery (Hassani et al., 2012). In a randomized controlled trial, patients injected with MSCs showed significant improvement in lower extremity motor function recovery (Chung et al., 2021). All of these proved that stem cells administration can improve ischemia stroke significantly and the therapeutic window is wide, suggesting that stem cells may be a potential clinical therapy for ischemia stroke, and multiple kinds of clinical trials based on stem cells are in progress (Supplementary Table S2).

### Molecular Mechanisms of Stem Cell Therapy

The most widely accepted mechanism of NSC-induced neuroprotective effects after stroke is *via* replacing the apoptosis cells. On the one hand, the transported NSCs could differentiate into neurons, which directly replaced the damaged neurons in the infarct area; on the other hand, the NSC-derived astrocytes would hypertrophy and form a glial scar which would block regenerated areas from ischemic cascade, preventing a new wave of damage, and at the same time secreting some growth factors, which can improve the microenvironment in the stroke area and reorganize the local loop to promote the neurovascular recovery (Gong et al., 2020). In addition, the newly formed oligodendrocyte would attach to neurons and warp them, thus forming myelin sheaths and promoting the recovery of rapid signal transmission; besides, NSC-derived oligodendrocytes helped to reduce blood–brain barrier leakage by upregulating β-catenin (Wang et al., 2020a). It showed that when injecting NSCs into the tract area, 20–80% of cells could differentiate into different types of neurons in different stages, such as general neurons, mature neurons, medium spiny projection neurons, and dopaminergic neurons (Zhou et al., 2019).

Meanwhile stem cells also have essential functions in immunomodulation. After injection, MSCs would migrate to the site of injury and polarize microglia into M2 phenotype, which played a role in anti-inflammation by secreting IL-4 and IL-10 (Li et al., 2018). Compared with the control group, the pro-inflammatory cytokines such as TNF-α, IL-6, and IL-1β decreased obviously, while activation of microglial increased after NSCs treatment (Eckert et al., 2015). In addition, MSCs could inhibit T cell proliferation by decreasing IFN-γ production and arresting T cell at G_1_ phase as well as exhibiting activated regulatory T-cells, an anti-inflammation T cell subgroup, resulting in increased IL-6 secretion and decreased TGF-β secretion (Neal et al., 2018). What is more, MSCs coculture strongly inhibited the initial differentiation of monocytes to dendritic cells (DCs) by increasing the secretion of IL-6 and enhanced the proliferation of DCs (Jiang et al., 2005; Yuan et al., 2019).

Apart from its anti-inflammation effects, trophic factors released by stem cells autocrine and paracrine also have strong neuroprotection effects, such as the brain-derived neurotrophic factor (BDNF), ciliary neurotrophic factor (CNTF), nerve growth factor (NGF), and VEGF. These trophic factors play a role in mediating cell proliferation and differentiation, for example, cocultured with MSCs leading to glial cells proliferation and differentiation, mostly due to BDNF and GDNF secretion (Pöyhönen et al., 2019; Tobin et al., 2020). Also, functional recovery was found with increased BDNF and VEGF in all stroke patients in a randomized MSC-treated trial, suggesting that trophic factors are important for the recovery of nerve function (Singh et al., 2020). When mice were treated with NSCs after MCAO, expression of BDNF increased which in turn promoted the maturation of neurons. The formation of synapse in the infarct area as well as increasing Vascular cell adhesion protein 1 (VCAM-1) and the macrophage colony-stimulating factor (MCSF) level, thus promoting chemokine secretion and migration (Tan et al., 2021).

Apart from MSCs and NSCs, there are many other cells showed a protective effect on stroke treatment, such as human umbilical cord blood cells, embryonic stem cells, endothelial progenitor cells, neural progenitor cells, induced pluripotent stem cells, and hematopoietic stem cells, all of which have multipotent abilities that could differentiation into different types of cells to replace the damaged one. In addition, these cells would secrete various neural trophic factors and anti-inflammatory factors as well as promoting angiogenesis which help with the recovery for ischemic stroke.

### Current Limitations for Stem Cell Therapy

As for stem cell therapy, an incipient therapy, there are still many factors limiting their clinical application, such as immune transplant rejection, side effects, unknown mechanisms, and the risk of tumor formation or the promising sources to obtain cells (Table 1). Firstly, the source of the cells is a big problem for patients. For patient’s treatment, they need a large number of cells, although MSCs can be derived from umbilical cord and adipose tissue, bone marrow is the most common source used in clinical trials, and large amount of bone marrow once from stroke patients is difficult to access. Cells such as NSCs and embryonic stem cells, mostly isolated from placenta or spinal cord, have ethical issues. Apart from that, stroke patients are weak and it is difficult to get transplantation cells from them and injection of cells from other people or species may cause organ rejection; as reported by Donders et al. (2015), *in vitro* licensed MSCs led to a fast rejection and enhanced immunogenicity. Another research also found that intracerebral transplantation of human NSCs into rats caused severe rejection (Jablonska et al., 2010). In addition, cell transplantation may cause tumor formation for its pluripotent, as indicated in research, two of nine mice formed tumors after NSCs injection (Fukuda et al., 2006).

**TABLE 1:** The challenge and research progress for therapies of stroke.

Therapy	Advantages	Current limitation	Structure transformation	Drug combination
Stem cell	Highly multipotent, differentiate into various types of neural cells such as neurons, oligodendrocytes, and astrocytes; autologous leads to low immunoreactivity	Risk of tumor formation; allogeneic may have immune reject; low infraction targeting; difficult to get large amount; more studies need to be done to confirm dosage, administration methods, and time window	Increase infraction targeting: ferrimagnetic iron oxide nanochains or ECM hydrogel delivery; decrease tumor formation: co-deliver with super-paramagnetic iron oxide nanoparticles or PEGylated super-paramagnetic iron oxide nanoparticles to increase neuron-like differentiation; decrease immune reject: Overexpression of IL-4/IL-10 in MSCs	Extend therapeutic window, inhibit side effects, and enhance therapeutic effects: tPA, minocycline, Niaspan, TCM, TMP, EPO, NBP, Sodium Ferulate, cyclosporin A, propranolol, and chlorzoxazone
Exosomes	The lipophilicity makes it easy to cross BBB; contain various cargos which plays important role for cellular interactions; no immune rejection	Production costs much and takes a long time; hard to distinguish the efficiency for itself and cargos; more studies need to be done to confirm dosage, administration methods, and time window	Selective expression of cargos: overexpress miR-25-3p, miR-21-5p, miR-873a-5p, miR-124, miR-193b-3p, and miR-132-3p; inhibit miR-206 or EP4	Enhance therapeutic effects: rosuvastatin
Traditional Chinese medicine	Wide range of herbal spices and can combine with different types of medicines to have multiple functions; no chemical synthesis is required	Specific mechanism is unsure; the quality of each TCM is different; inner toxicity; high incidence of liver and kidney injury	Analyze the structure of the active ingredient, modify and synthesize it artificially, or semi synthesize compounds such as NBP, PNS, and Sal	Extend therapeutic window, inhibit side effects, and enhance therapeutic effects: tPA with T541, MSCs with TMP, clopidogrel with PNS/Danshen/Ginkgo, Nimodipine with Ginkgo, and resolvin D1 with Xuebijing

The dose for injection is another problem that confused many people since the cell death caused by injection or inflammatory clear is not sure, also the transplantation methods, the severity of disease and the conditions of patients all affect the dosage of transplantation. A dose-dependent study was evaluated by intravenously injecting a low dose (1 x 10^4^) or a high dose (1 x 10^5^) MSCs in mice for 48 h after MCAO, and the results showed that the high dose group had better motor recovery and less infarction area (Tanaka et al., 2018). In contrast, another group of mice treated with three different MSCs dosages (2.4 x 10^7^, 2.4 x 10^8^, and 4.4 x 10^8^) showed improved recovery, but there was no difference between these doses (Hashemi et al., 2008). It is difficult to conclude which kind of dosage fits patients best, one dose injection may be the best therapeutic way for acute stage, as for long-term recovery, low-dose multiple injections for a period of time may be better.

What is more, the transplantation methods are important too. Intravenous is the most widely used method in preclinical studies and it can promote recovery significantly; however, in most researches, intravenous in animal models could cause lung, liver, and spleen embolisms like pulmonary embolisms and diffuse alveolar hemorrhages, which happened in patients as demonstrated by two cases with venous clots at the proximal end of the puncture site (Oeller et al., 2018). Apart from that, many studies showed that the majority of cells were trapped in the peripheral organs such as the lung, spleen, and liver because of blood circulation and only a few of them got to the infarction area and differentiated into neuronal cells and glial cells (Ankrum and Karp, 2010). On the contrary, other people found that distribution in other organs is rare (Moon et al., 2019). Another widely used method is intracerebral transplantation, directly injecting compounds into brain; however, it may damage brain tissue and cause some unexpected side effects. For example, in a clinical trial, researchers observed 17 serious adverse events in 11 patients including partial seizures, sepsis, subdural hemorrhage, and vomiting after NSCs intracerebral transplantation (Muir et al., 2020). Nowadays, lumbar puncture, arterial infusion, and intranasal have also become viable methods but fewer people use these methods and their clinical utility is still uncertain.

Also, the timing of injection is important, which depends on the state of the compounds and the stage of the diseases. Cell’s viability will decrease when they were cultured for a long time or passaged too many times. At the first few hours or days, patients are under unstable phase and it is difficult to do injection at this time. In clinical studies, transplanting cells 24 h to 3 months after stroke both promote neurological function recovery, so the certain time window for transplantation is unsure (Zheng et al., 2018).

### Improvements for Stem Cell Therapy

As shown in multiple studies, a large number of cells die or migrate to other organs leading to low therapeutic efficiency, so many researchers tried to get better infarction targeting by changing the cell structure (Table 1). Zhang et al. (2019) designed fabrication of magnetosome-like 1D ferrimagnetic iron oxide nanochains (MFIONs) which increased the MSCs delivery efficiency to 2-3 folds and decreased the mortality of stroke. Other researchers tried using poly (ethylene glycol) (PEG)–conjugated phospholipid (PEG-lipid) as a ligand and conjugated with E-selectin-binding oligopeptide (ES-bp) which improved the cell attachment in active endothelium cells in the damage area (Noiri et al., 2019). Others used a PEG microsphere to encapsulate NSCs and suspended these microspheres in the extracellular matrix (ECM) hydrogel, then injected the modified cells into mice, this ECM hydrogel delivered NSCs to the infarct area 5-fold than the control group (Ghuman et al., 2021).

For another perspective, some people tried to enhance cell differentiation or the secretion of trophic factors to improve their therapeutic effect. For example, Lin et al. (2021) co-delivered super-paramagnetic iron oxide nanoparticles (SPIO), *Pnky* siRNA, and antisense oligonucleotides (ASO) into NSCs, guiding NSCs to directly differentiate into neurons, at the same time it made NSCs traceable. When treated BMSCs with PEGylated super-paramagnetic iron oxide nanoparticles (IONP), which enhanced expression of angiogenic, neuroprotective, and anti-inflammation factors in the MSCs and also increased the exosomes secreted by MSCs to the ischemia lesion by 5.1 times, which together promoted the neurovascular unit recovery (Kim et al., 2020). What is more, transducing NSCs with Neurogenin 2 (NEUROG2) adenoviral significantly increased the level of CNTF and NTF3 around 3 times, which further promotes neurite growth and axonal sprouting (Lee et al., 2017).

Since most cells are derived heterogeneously, immune resistance kills some of them which lead to cell death and less efficiency. Consequently, researchers focus on structure transformation to reduce inflammatory damage. Overexpression of IL-4 in MSCs before intrahippocampal injections obviously induced the M2 polarization of microglia and an anti-inflammation status as well as the activation of macroglia, leading to better motor function recovery and less MSCs death (Enam et al., 2020). In the same way, delivering recombinant IL-10 alongside MSCs increased anti-inflammation effects as indicated by fewer astrocytes and glial scar formation (Peruzzaro et al., 2019). Additionally, other transformations had been studied like iron oxide–labeled MSC that could be tracked by MRI which led to MSC visualization in brain, seeding NSCs onto poly (glycolic acid)-based scaffold enhanced angiogenesis (Mishra et al., 2018), and more and more methods were carried out to overcome existing limitations.

Since there is no specific drug used for ischemic stroke, many adjuvant drugs, such as antiplatelet drugs, anticoagulants, and antibiotics are used for stroke treatment. With the development of cell therapy, combination therapy has proved to be more effective. Niaspan helped to lower LDL, when used for 14 consecutive days after MSC injection in MCAO mice, and it decreased the incidence of cerebral hemorrhage caused by MSCs and at the same time increased the motor function recovery (Yan et al., 2013). As for antibiotics, minocycline had an anti-inflammatory effect, when combined with NSCs, and it could inhibit the astrocytic differentiation and restore the potential to differentiate into neurons and oligodendrocytes, and thus enhanced the cell replacement in infarction volume and promoted the recovery of stroke (Vay et al., 2016). What is more, propranolol, a b-Adrenergic receptor antagonist, together with MSCs could augment neurogenesis and inhibit microglia activation for both short and long term, without accumulation of side effects (Kota et al., 2016). Moreover, treated MSCs 3 days post MCAO with erythropoietin got better motor recovery and decreased the infarction volume, which meant that combination therapy could extend the therapeutic window for cells (Larpthaveesarp et al., 2021). All these results indicated that combination cells with other adjuvant drugs could promote the efficacy of drugs, reduce side effects, and prolong the drug treatment window.

Meanwhile, it seems that cell therapy combined with tPA attenuating side effects for tPA effectively. Recent study showed that injecting MSCs 30 min later than tPA treatment diminished the ratio of hemorrhage with better functional recovery by inhibiting MMP9 activation (Nakazaki et al., 2017). What is more, injecting tPA 6 h post stroke would cause severe neurovascular damage; however, tPA treatment followed by NSCs transplantation 18 h later could reverse the damage by inhibiting BBB leakage and anti-inflammatory (Boese et al., 2020).

## Exosome Therapy

Exosomes are lipid bilayer vesicles with a diameter of 30–150 nm, released by cells in the living systems followed by stimulation such as oxidative stress, antigens, or endotoxins. Late-stage endosomes inward bud and form multivesicular bodies, these multivesicular bodies fuse with the plasma membrane, releasing the internal vesicles into the extracellular space, which become exosomes (Hessvik and Llorente, 2018). For its lipophilicity, exosomes are able to pass through the BBB and are present in blood and cerebrospinal fluid. Besides, there are many cargos in the exosomes (proteins, nucleic acids, lipids, miRNAs, DNA, peptides, and cytokines) that play an important role in mediating cellular interactions (Mateescu et al., 2017). It has been reported that exosomes derived from MSCs could get a protective effect on stroke, for its easy and longtime storage (restore for 6 months at -80°C), no immune rejection and high concentration in body fluids, exosomes have become a new research trend.

### Molecular Mechanisms of Exosome Therapy

The most widely studied exosomes were MSC-derived exosomes. Xin et al. found that tail vein injection of 100 μg MSC-derived exosomes 24 h post MCAO surgery significantly improved neurologic outcome and ameliorated lesion volume, at the same time promoting neurovascular plasticity with increased neurite remodeling, newly formed neuroblasts and endothelial cells (Xin et al., 2013). Others also discussed the long-term neuroprotection associated with improved angiogenesis after injecting MSC-derived exosomes 1–5 days after surgery (Doeppner et al., 2015). Enhanced neurological outcomes from MSC-derived exosomes are comparable to those observed with MSCs treatment, suggesting that exosomes may contribute to ischemic stroke therapy.

Several preclinical studies indicated that MSC-derived exosomes reduced inflammation. Exosomes from MSCs reduced neuronal injury by inhibiting the brain infiltration of macrophages, lymphocytes, leukocytes, and especially polymorphonuclear neutrophils (PMN), which showed benefit to stroke, and also depletion of PMN ameliorated the effects of exosomes on other immune cells such as B cells and T cells (Dabrowska et al., 2019). Compared with the control group, there were fewer pro-inflammation cytokines like IL-6 and IL-1α in the exosome’s treatment group, with fewer microglial, leukocytes, and cytotoxic T cells (Wang et al., 2020b). Improved functional recovery, neurogenesis, and vasculogenesis were found after MSC-secreted exosomes treatment, and all these were caused by inflammation response to TNF-α and IL-1β and microglial activation both *in vivo* and *in vitro* (Geng et al., 2019).

Apart from MSC-derived exosomes, exosomes from other cells also play a role in stroke therapeutic. Administration of exosomes obtained from NSCs in pig led to obvious elimination in ischemic lesion, less brain swelling, and edema with improved white matter integrity and behavior (Webb et al., 2018). Apart from that, the resistance of cerebral organoids exposed to OGD was enhanced by neural progenitor cells (NPCs)–derived exosomes. Mice treated with exosomes obtained from NPCs had reduced motor coordination impairment, increased long-term neuroprotection, cell proliferation, and axonal plasticity through enhancing peripheral B cell’s and T cell’s anti-inflammation effects (Zheng et al., 2021). Considering the important effect of macrophages in stroke immune regulation, exosomes secreted from macrophages also have some effects on stroke therapeutic. Injection of M2 phenotype macrophage-derived exosomes for seven consecutive days after MCAO surgery, the glial scar formation, astrocyte activation, and proliferation were inhibited visibly. It also enhanced the transition of astrocytes to neural progenitors through blocking the STAT3 pathway, and the effect may be caused by upregulating miR124 and its downstream protein ubiquitin-specific protease 14 (USP14) (Li et al., 2021). In addition, exosomes from LPS-stimulated macrophages promoted the conversion of microglial from pro-inflammation M1 to anti-inflammation M2 phase, thus increasing the survival of neuron cells. It also had a protective effect on the *in vivo* stroke model (Zheng et al., 2019). Astrocytes are activated during stroke and play a protective role for infarction and neurovascular recovery *via* secreting nutritional factors, as indicated by Pei et al.; astrocyte-derived exosomes inhibited the apoptosis and pro-inflammation factors in neuron cells, also autophagy-associated proteins such as P62 and LC3 were increased after exosomes in stroke model mice (Pei et al., 2019). Endothelial progenitor cells (EPCs) had the effect of promoting vascular repair and regeneration, and exosomes derived from EPCs also had been demonstrated to reduce ischemic-induced brain injury, accompanied by improved microvascular density, cerebral blood flow, and angiogenesis through upregulating VEGFR2 (Wang et al., 2020c). These data suggested that exosomes secreted by different cell types could engage in brain remodeling by neurovascular unit regeneration during stroke recovery.

### Current Limitations for Exosome Therapy

The source is still a big problem for exosome therapy. Treatment of astrocytes with high KCl concentrations led to the secret of exosomes, while NaCl could regulate the release of exosomes from oligodendrocytes but producing and isolating large-scale exosomes cost much and take a long time (Nikfarjam et al., 2020). Apart from that, it is difficult to make a standard for exosomes in use since there are no rules to distinguish whether each exosome is useful because the size, cargos, and markers are different from individual exosomes and difficult to test the cargos or markers inside each exosome. Meanwhile, there are too many cargos in an exosome, some of which play therapeutic effects, while others may not be useful for different organs. In addition, the interaction between exosomes and other cells is complicated; exosomes from macrophage and T cells mediate the transfer of CCR5 and CXCR4 to other cells and turn these cells vulnerable to human immunodeficiency virus-1 (Madison and Okeoma, 2015). What is more, other side effects have shown in exosomes therapeutic, for example, exosomes from hypothalamic stem cells affect the aging speed in mice (Zhang et al., 2017b). Systemic inflammation induces the production of exosomes into cerebrospinal fluid and are endocytosed by astrocytes and microglia, upregulating inflammatory in brain, and blocking of exosomes secretion inhibit brain inflammation (Balusu et al., 2016).

Since exosomes are derived from cells, they have some limitations in common; the dose for injection, the therapeutic window, and the administration methods still need to be determined before using in clinical trials. Some studies reported that the number of exosomes derived from MSCs in the infarction lesion and the functional recovery increased in a dose-dependent manner and the distribution in other organs is rare (Moon et al., 2019). In contrast, others found that only low dose exosomes treatment had neuroprotection effect rather than a high dose and organs such as the liver, spleen and lung had a large number of exosomes (Otero-Ortega et al., 2020). More preclinical investigations in different species may need to overcome these limitations.

### Improvements for Exosome Therapy

Since researches work on exosomes began late, fewer people use it for drug combination. Study reported that MSC-derived exosomes taking with rosuvastatin, a cholesterol-lowering drug, for 7 days significantly decreased the infarction volume than monotherapy by decreasing the number of astrocytes and inflammasome NLRP3 (Safakheil and Safakheil, 2020). In addition, modification methods are also relatively limited, and cargo-like miRNA is most widely used in exosomes modification. Exosomes derived from miR-17-92-clustered MSCs significantly improved the neurite remodeling, neuronal dendritic plasticity, neurogenesis, and oligodendrogenesis *via* activating the PI3K/AKT/mTOR pathway (Xin et al., 2017). The infarct area and neurological recovery were increased in mice treated with exosomes obtained from miR-25-3p-overexpressed MSCs because of the enhanced autophagic flux, p53, and the BCL2-interacting protein 3 (BNIP3) pathway, and in contrast, loss of miR-25-3p in MSC-derived exosomes diminished neuroprotection induced by exosomes obviously (Kuang et al., 2020). MiR-21-5p was highly expressed at the MSC-derived exosomes when applied in stroke. The knockdown of miR-21-5p in exosomes accelerated neuron cell death in the *in vitro* stroke model *via* inhibiting the PTEN/Akt pathway (Gao et al., 2020). Moreover, astrocyte-derived exosomes enriched with miR-873a-5p inhibited the formation of M1 macrophage in the infarction area by downregulating NF-κB, thus fewer pro-inflammation factors are produced (Table 1).

## Conclusion and Perspective

**FIGURE 2:**
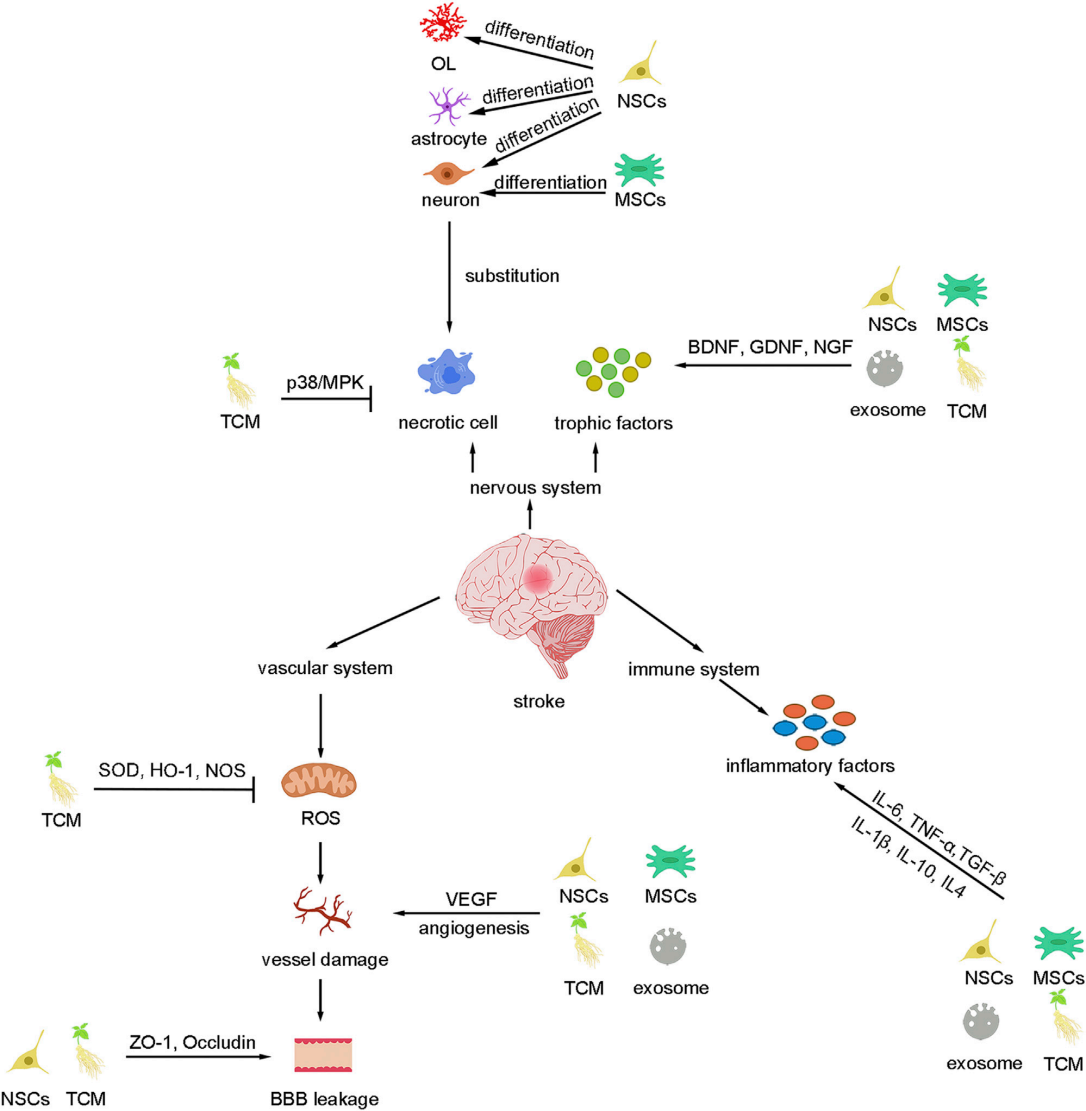
Summary of basic mechanisms underlying different therapies for stroke. These therapies can inhibit cell death and replace the necrotic cells as well as secret trophic factors to improve the nervous system. Besides, these therapies protect the vascular system by inhibiting ROS, BBB leakage, angiogenesis, and inflammation.

Stroke is a disease with a high incidence, mortality, and disability rate, the only drug approved by FDA is tPA, but the time window and the risk of hemorrhage limit its usage. The neurovascular unit is important for central nervous system homeostasis, but till now, no drug has been approved to improve the neurovascular unit after stroke. In the review, we discussed the potential therapeutic agents for stroke neurovascular treatment including Traditional Chinese medicine (ginkgolides, Sal, and PNS), stem cell therapy (MSCs and NSCs), and exosomes. We briefly talked about the potential mechanism for these agents which mediating the neurovascular unit recovery, such as anti-apoptosis, anti-inflammation, neural plasticity, and angiogenesis (Figure 2). Moreover, we compared the current limitations for these therapies emphatically. Based on these limitations, many researchers tried structure transformation or drug combination to increase the survival and migration of cells, decrease the side effects, enhance the therapeutic effects and expand the treatment window (Table 1). It seems that there are some overlaps in the effects of these drugs and the mechanism for TCM is not fully investigated, we cannot conclude that a single treatment of these drugs could be sufficient for clinical neurovascular achievement.

However, there are still some limitations for these agents we have mentioned since the side effects and the unknown time window, dosage, and injection methods restrict the usage. Additional experimental considerations and clinical studies are required before clinical application. For stem cells and exosomes therapy, how to get a large number of cells/exosomes, how to increase the survival of most engrafted cells, how to guide most cells/exosomes to the infarction lesion, and how to detect the distribution of cells/exosomes without any tissue damage, all of these questions need to be resolved in clinical trials. Moreover, almost all the experiments are done on healthy animals, and some highly relevant complications such as diabetics, hypertension, hyperlipidemia, and heart disease may need to be considered since they can affect the formation of stroke and treatment. Besides, gender and age differences are also of concern in the investigation. As the majority of ischemic stroke patients could not be treated in time, safe and effective modifications in order to expand the therapeutic window of drugs may need to be carried out. Monotherapy may only play a therapeutic role in one aspect, combination use of drugs is required, so pay attention to the interaction between drugs.

In conclusion, there are many potential therapeutic agents for stroke treatment that are useful in preclinical investigations for recovery of the neurovascular unit, but most of them are still under clinical trials since their efficacy and side effects are still unknown in patients. Further investigations are needed to demonstrate the efficacy and safety of these agents, leading to the completion of the process from the bench to bedside. What is more, considering the complications and unexpected epidemics such as COVID-19, there is still a demand to develop new compounds for lacking of drugs to treat stroke. Meanwhile, it will be vital to overcome resistance and extend the clinical application of conventional drugs.
