# Supplementary material for: Challenges and Improvements of Novel Therapies for Ischemic Stroke
Source: Front Pharmacol. 2021 Sep 30;12:721156. doi: 10.3389/fphar.2021.721156 (PMC8514732; doi:10.3389/fphar.2021.721156)
Supplement: Supplementary file 1 [file DataSheet1.docx]

**Supplementary Tables**

Supplementary Table 1 Traditional Chinese Medicine in stroke clinical trails

| agent | Active  ingredient | trial | phase | current  status | study result |
| --- | --- | --- | --- | --- | --- |
| Danhong Injection | Salvia miltiorrhiza, carthamus tinctorius | A Clinical Trial of Danhong Injection in Treating Acute Ischemic Stroke | phase 4 | active | infarct volume reduction, Neurological deficit correction, reverse the abnormal arterial, inhibit β-adrenergic pathway and protein kinase C |
| Ginkgo Diterpene Lactone Meglumine Injection | Ginkgolides | Ginkgo Diterpene Lactone Meglumine Injection on Platelet Reactivity in Acute Ischemic Stroke | phase 4 | completed | recovery of patients in the subacute period, reduce inflammatory cytokines and Coagulation Factor III, up-regulate anti-inflammatory cytokines |
| Ixeris of sonchifolia Hance | Ixeris of sonchifolia Hance | Heat-clearing and Blood-activating Chinese Medicinal Components in Acute Cerebral Infarction | ND | recruiting | improve microcircula |
| Panax notoginseng saponins | Panax notoginseng saponins | Heat-clearing and Blood-activating Chinese Medicinal Components in Acute Cerebral Infarction | ND | recruiting | reduce infarction area and increase Nissl bodies, neuron protection |
| Granules of Shengdi Dahuang Decoction | rehmanniae praeparatum, rheum officinale, Scutellaria baicalensis | Clinical Evaluation of Shengdi Dahuang Decoction in the Treatment of Acute Hemorrhagic Stroke | phase 4 | recruiting | improve anti-inflammatory effects and neurological function, inhibit microglial activation |
| AngongNiuhuang | Calculus bovis | Efficacy and Safety of AngongNiuhuang Pill in Patients With Acute Ischemic Stroke | phase 3 | not yet recruiting | better neurological score, less infarct area, cell damage and apoptosis |
| Huatuo Zaizao Pills | Ligusticum wallichii | Efficacy Study of Huatuo Zaizao Pills in Improving of Neural Function in Acute Ischemic Stroke | phase 4 | recruiting | promote functional recovery and generation of new neurons, enhance the expression of BDNF |
| Xingnaojing injection | musk, borneol | Xingnaojing for Mild-to-severe Acute Ischemic Stroke | phase 4 | recruiting | decrease cerebral infarct area, improve neurological scores and morphological changes, inhibit the inflammatory response |
| Buqitongluo | Angelica sinensis, Salvia miltiorrhiza, Caulis Polygoni Multiflori | BuqitongluO Granule for Qi Deficiency and Blood Stasis Syndrome | phase 2 | recruiting | improved neurological score |
| Yiqitongluo granule | Angelica sinensis, Salvia miltiorrhiza, Caulis Polygoni Multiflori | Effectiveness and Safety of Yiqitongluo Granule for Stroke | phase 4 | completed | improve neurological scores, regional cerebral blood flow |
| Qizhitongluo Capsule | Salvia miltiorrhiza, Astragalus membranaceus | Efficacy and Safety of Qizhitongluo Capsule in the Recovery Phase of Ischemic Stroke | phase 4 | completed | improve lower limb motor recovery |
| Naoxintong Capsule | Astragalus membranaceus, Salvia miltiorrhiza, Angelica sinensis | Curative Efficacy of Secondary Prevention for Patients With Ischemic Stroke Through Syndrome Differentiation of TCM | phase 4 | completed | improved neurological score |
| Astragalus membranaceus | Astragalus membranaceus | Randomized, Double Blind, Placebo Control Trial to Evaluate the Efficacy of Astragalus Membranaceus in the Patients After Stroke With Fatigue | phase 4 | recruiting | attenuate the neurological deficit, reduce cerebral infarction and neuronal apoptosis |
| SaiLuoTong capsule | Panax Notoginseng Saponins | The Clinical Trial of Chinese Herbal Medicine SaiLuoTong Capsule | phase 2 | active | memory recovery, reduce the astrocytic reaction |
| Shuxuetong Injection | Hirudo, lumbricus | A Registry Study on Shuxuetong (a Chinese Medicine Injection) Used in Twenty Hospitals | phase 4 | completed | increase cell viability, inhibit production of reactive oxygen species and mitochondrial superoxide |
| ginsenoside-Rd | ginsenoside-Rd | Ginsenoside-Rd for Acute Ischemic Stroke | phase 2 | completed | neuroprotective efects, inhibit calcineurin activity |
| Sanchitongshu | Pantotriol saponins | Sanchitongtshu Plus Asprine for Minor Ischemic Stroke or Transient Ischemic Attack: A Randomized Double-blind Study | ND | not yet recruiting | ameliorate neurological deficit |
| Dengzhan Shengmai capsule | Erigeron breviscapus, ginseng, Schisandra chinensis | The Secondary Prevention Trial for Ischemic Stroke With DengzhanShengmai Capsule | phase 4 | completed | improve neurological function |

From information available at ClinicalTrials.gov (see also https://clinicaltrials.gov)

Supplementary Table 2 MSCs and NSCs transplantation in stroke clinical trails

| Country | Phase | Current status | Cell Source/  Autologous or allogeneic | Doses | Route | Time from  stroke onset | Sample cases |
| --- | --- | --- | --- | --- | --- | --- | --- |
| France | Phase 2 | Completed | MSCs/Autologous | ND | IV | <6 months | 31 |
| Malaysia | Phase 1/2 | Withdrawn | MSCs/Allogeneic | 2 x 10^6^ | IV | <10 days | 0 |
| USA | Phase 1/2 | Completed | BMSCs/Allogeneic | 0.5-1.5 x 10^6^ | IV | >24 weeks | 38 |
| Malaysia | Phase 2 | Unknown | BMSCs/Autologous | ND | IV | 2-8 weeks | 50 |
| China | Phase 1/2 | Unknown | BMSCs/Autologous | 2.5 x 10^6^ | IV | <1 week | 20 |
| Spain | Phase 2 | Completed | ADSCs/Allogeneic | 1 x 10^6^ | IV | <2 weeks | 19 |
| China | Phase 1 | Unknown | BMSCs/Autologous | 2-4 x 10^6^ | IC | 3-60 months | 30 |
| Korea | Phase 3 | Unknown | MSCs/Autologous | ND | IV | <90 days | 60 |
| USA | Phase 1 | Withdrawn | BMSCs/Allogeneic | ND | IV | ND | ND |
| USA | Phase 1/2 | Withdrawn | BMSCs/Allogeneic | ND | IV | 3-10 days | 0 |
| China | Phase 1 | Unknown | BMSCs/Autologous | ND | IV | 6-60 months | 40 |
| China | Phase 2 | Unknown | UMSCs/Allogeneic | 2 x 10^7^ | IV | <12 weeks | 2 |
| France | Phase 2/3 | Withdrawn | ADSCs/Allogeneic | ND | IV | 1-4 days | 0 |
| China | Phase 1/2 | Suspended | UMSCs/Allogeneic | ND | IV | ND | 40 |
| China | Phase 1 | Suspended | UMSCs/Allogeneic | 0.5-1 x 10^6^ | IV | <2 weeks | 40 |
| Netherlands | Phase 1/2 | Recruiting | BMSCs/Allogeneic | 5 x 10^7^ | Nasal | <7 days | 10 |
| USA | Phase 1 | Completed | BMSCs/Allogeneic | 1-5 x 10^6^ | IV | ND | 9 |
| Iran | Phase 1/2 | Recruiting | MSCs/Allogeneic | ND | IV | 1 month | 5 |
| USA | Phase 1/2 | Recruiting | ADSCs/Autologous | 2 x 10^8^ | IV | <6 weeks | 24 |
| China | Phase 1/2 | Recruiting | UMSCs/Allogeneic | 2 x 10^6^ | IV | <7 days | 120 |
| China | Phase 1 | Recruiting | UMSCs/Allogeneic | ND | IV | ND | 9 |
| Spain | Phase 2 | Recruiting | ADSCs/Allogeneic | 1 x 10^6^ | IV | <4 days | 30 |
| China | Phase 1 | Recruiting | UMSCs/Allogeneic | ND | IA+IV | ND | 14 |
| China | Phase 1/2 | Net yet recruiting | MSCs/Allogeneic | 0.5-2 x 10^6^ | IV | ND | 60 |
| UK | Phase 1 | Active | CTX0E03/allogeneic | 0.5-2 x 10^6^ | IA | <4 weeks | 12 |
| UK | Phase 2 | Completed | CTX DP/allogeneic | 2 x 10^7^ | IA | 2-3 months | 23 |
| China | Phase 1 | Unknown | NSI-566/allogeneic | 1.2-8 x 10^7^ | IA | ND | 18 |
| USA | Phase 2 | Active | CTX0E03/allogeneic | 2 x 10^7^ | IA | ND | 130 |
| USA | Phase 1/2 | Recruiting | NR1/allogeneic | ND | IA | ND | 30 |

From information available at ClinicalTrials.gov (see also https://clinicaltrials.gov)
